# Structure–Activity Relationship of RGD-Containing Cyclic Octapeptide and αvβ3 Integrin Allows for Rapid Identification of a New Peptide Antagonist

**DOI:** 10.3390/ijms21093076

**Published:** 2020-04-27

**Authors:** Aaron Silva, Wenwu Xiao, Yan Wang, Wei Wang, Heng Wei Chang, James B. Ames, Kit S. Lam, Yonghong Zhang

**Affiliations:** 1 Department of Chemistry, The University of Texas Rio Grande Valley, Edinburg, TX 78539, USA; aaron.silva01@utrgv.edu; 2 Department of Biochemistry and Molecular Medicine, University of California, Davis Cancer Center, Sacramento, CA 95616, USA; wxiao@UCDAVIS.EDU (W.X.); kslam@ucdavis.edu (K.S.L.); 3 CSBio Company Inc., Menlo Park, CA 94025, USA; yanwx88@gmail.com (Y.W.); wei.wang@csbio.com (W.W.); chang@csbio.com (H.W.C.); 4 Department of Chemistry, University of California, Davis, CA 95616, USA; jbames@ucdavis.edu

**Keywords:** integrin αvβ3 antagonists, RGD peptides, structure–activity relationship, in silico screening, in vitro binding

## Abstract

The αvβ3 integrin, a receptor for many extracellular matrix proteins with RGD-sequence motif, is involved in multiple physiological processes and highly expressed in tumor cells, therefore making it a target for cancer therapy and tumor imaging. Several RGD-containing cyclic octapeptide (named LXW analogs) were screened as αvβ3 antagonists with dramatically different binding affinity, and their structure–activity relationship (SAR) remains elusive. We performed systematic SAR studies and optimized LXW analogs to improve antagonistic potency. The NMR structure of LXW64 was determined and docked to the integrin. Structural comparison and docking studies suggested that the hydrophobicity and aromaticity of the X7 amino acid are highly important for LXW analogs binding to the integrin, a potential hydrophobic pocket on the integrin surface was proposed to play a role in stabilizing the peptide binding. To develop a cost-efficient and fast screening method, computational docking was performed on LXW analogs and compared with in vitro screening. A consistency within the results of both methods was found, leading to the continuous optimization and testing of LXW mutants via in silico screening. Several new LXW analogs were predicted as the integrin antagonists, one of which—LXZ2—was validated by in vitro examination. Our study provides new insight into the RGD recognition specificity and valuable clues for rational design of novel αvβ3 antagonists.

## 1. Introduction

Integrin αvβ3 known as the vitronectin receptor is a member of the integrin superfamily and a heterodimeric transmembrane protein formed by non-covalent association of αv and β3 subunits. Each subunit consists of a large extracellular domain, a single transmembrane domain, and a short cytoplasmic domain, through which the integrin modulates bi-directional cell signaling over the plasma membrane [1]. As a cell surface receptor of the extracellular matrix (ECM), it binds a wide variety of ECM ligands with RGD motif implicated in many normal and pathological cell functions including cell survival, angiogenesis, tumor invasion, etc. [2]. Unlike other integrins ubiquitously expressed in adult tissues, αvβ3 is most abundantly expressed on angiogenic endothelial cells in pathological tissues [3]. In fact, the inhibition of αvβ3 has been widely used in clinical trials as anti-angiogenic therapy with growing interest in developing inhibitors specifically targeting αvβ3 integrin in the past decade [4].

RGD peptides are well-known to bind to the integrins including αvβ3 as revealed by the crystal structure of αvβ3 ectodomain in complex with the cyclic RGD peptide—cilengitide [5]—which provided the structural basis for development of αvβ3 antagonists. Targeting tumor cells or tumor vasculature by RGD-based strategies is a promising approach for delivering anticancer drugs or contrast agents for cancer therapy and diagnosis. RGD-based strategies include RGD peptide or peptidomimetic drugs, RGD-conjugates, and the grafting of the RGD peptide or peptidomimetic, as targeting ligand, at the surface of nanocarriers [6]. A series of RGD-containing cyclic octapeptides—LXW analogs—have been reported using the one-bead-one-compound (OBOC) combinatorial library technology (Table 1) [7,8]. LXW7 was identified as a leading ligand that binds specifically to αvβ3 integrin with a comparable binding affinity to those well-known RGD-cyclic pentapeptide ligands [7]. A further systematic optimization of LXW analogs was conducted and led to identification of several more potent LXW peptides [8]. One of the best ligands-LXW64 demonstrated 6-fold higher binding affinity than LXW7 and identified as the new lead [8]. However, the SAR remains elusive as these LXW analogs share similar structures but exhibit substantially different integrin binding affinities. Furthermore, this in vitro screening procedure requires considerable efforts such as synthesis of OBOC libraries, on-bead whole-cell screening assays, etc. which is often time-consuming and costly. To develop and apply a rapid, low-cost in silico screening method combining with selective in vitro validation would be a better way for this purpose. As a continuation of our previous efforts in developing αvβ3 antagonists, we herein introduce a combinatorial method, report identification of a new RGD-containing cyclic octapeptide against αvβ3 integrin. Its high binding affinity to the integrin has been validated using the competition binding assay on αvβ3 integrin-transfected cells (K562/αvβ3+).

## 2. Results

### 2.1. NMR Assignments of LXW64 and Verification of Disulfide Bond

**Figure 1:**
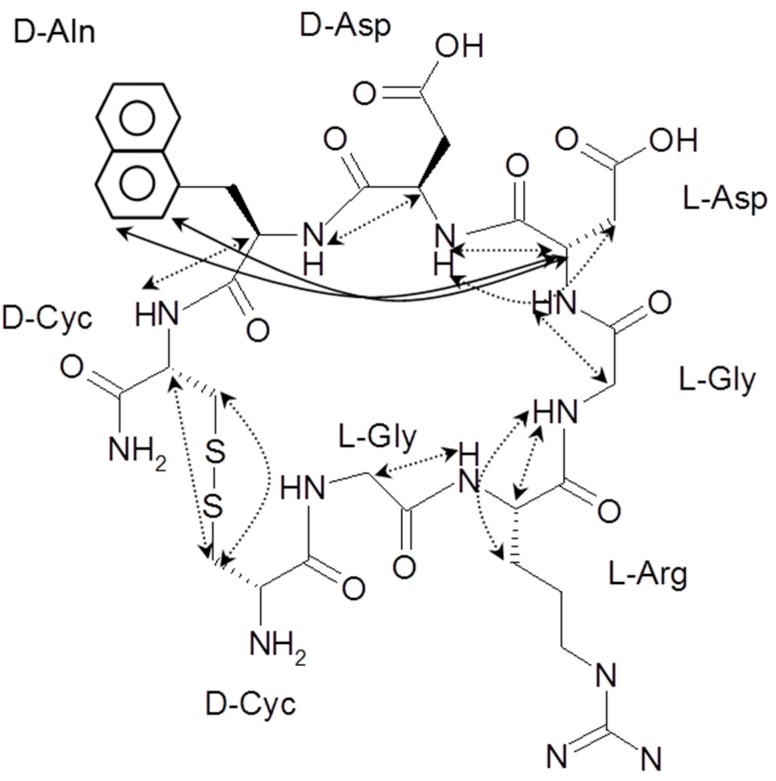
The observed NOEs of LXW64 in 2D 1H-1H NOESY (dashed lines for sequential NOEs; solid lines for long-range NOEs).

LXW64 (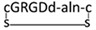) contains 8 amino acids including non-proteinogenic amino acid—3-(1-naphthyl)-D-alanine (D-Nal1). The ^1^H-coupling spin system for each residue type was unique and easily distinguishable from ^1^H-^1^H TOCSY, for example, the residue, arginine, was unambiguously assigned based on its unique ^1^H resonances of H^β^ and H^γ^ (1–2 ppm). The other three types of residues, two glycines, two aspartates, and two cysteines, were also identified and assigned with their H^N^, H^α^, and H^β^. The sequential assignment was then completed through the connectivity of NOEs observed between the amide protons in 2D NOESY (Figure 1). Thus, all ^1^H resonances were unambiguously assigned for LXW64. The proton assignments allowed unambiguous assignments of all proton-attached ^13^C resonances using ^1^H-^13^C HMQC spectrum, while the chemical shifts of non-pronated ^13^C were assigned from ^1^H-^13^C HMBC spectrum due to their long-range couplings with other assigned protons. Native abundance gradient ^15^N HSQC spectrum was obtained for LXW64 on the 600 MHz Bruker spectrometer. The high-signal-to-noise quality of this spectrum enabled unambiguous assignments for all ^15^N chemical shifts. Chemical shifts of all ^1^H, ^13^C and ^15^N were fully assigned for LXW64 peptide and are available as Supporting Information in Table S1. The intramolecular disulfide bond of the peptide was confirmed via the NOEs between two cysteines (Cys1 and Cys8). Moreover, the disulfide bridge connectivity was identified by MS and ^13^C^β^ chemical shifts of two cysteines [9].

### 2.2. Structure Determination of LXW64 and Structural Comparison with Other LXW Peptides

**Figure 2:**
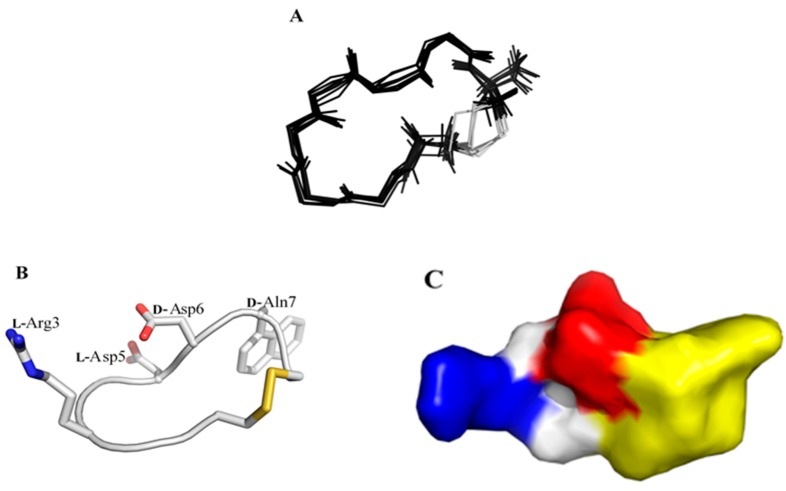
NMR-derived structures of LXW64. (**A**) An ensemble of 10 superimposed minimum energy structures. (**B**) Sticks representation of the energy-minimized average structure, in which *L-*Arg3, *L*-Asp5, D-Asp6 and D-Nal1 (X7) are labeled. (**C**) Representation of the molecular surface of LXW64, colored by electrostatic potential. Blue and red color represents positive (Arg) and negative (Asp) potential, respectively. The hydrophobic portion is highlighted in yellow.

All sequential NOEs between α proton of residue i (H^α^_i_) and amide proton of residue i+1 (H^N^_i+1_) were observed in 2D NOESY, as shown in the molecular structure of LXW64 (Figure 1). Interestingly, NOEs between H^α^_5_ and the aromatic side-chain protons (H^δ2^ and H^ε2^) of *D-Nal1* was observed, which is similar toLXW7 but missed in LXW7 isomer, i.e., LXW11 [8]. The NMR-derived distance constraints from 2D NOESY as well as the dihedral constraints from *J* coupling constants and carbon chemical shifts (Table 2), allow us to perform atomic resolution structure calculation. The final NMR-derived structures are illustrated in Figure 2 and summarized in Table 2. The 10 lowest-energy conformers when superimposed have an overall main chain root-mean-squared derivation (RMSD) of 0.34 ± 0.072 Å. The energy-minimized average structure of LXW64 is shown in Figure 2B (see for the structure coordinate (S2 LXW64 Coordinates)). The peptide adopts a bowl-shape and open circular structure with all side chains pointing toward the outside. The structure (Figure 2C) contains positively (*L*-Arg3) and negatively (*L*-Asp5 and *D*-Asp6) charged portions and hydrophobic moiety (*D*-Nal1), which may contribute to hydrophilic, electrostatic and hydrophobic interactions when binding to αvβ3 integrin.

**Figure 3:**
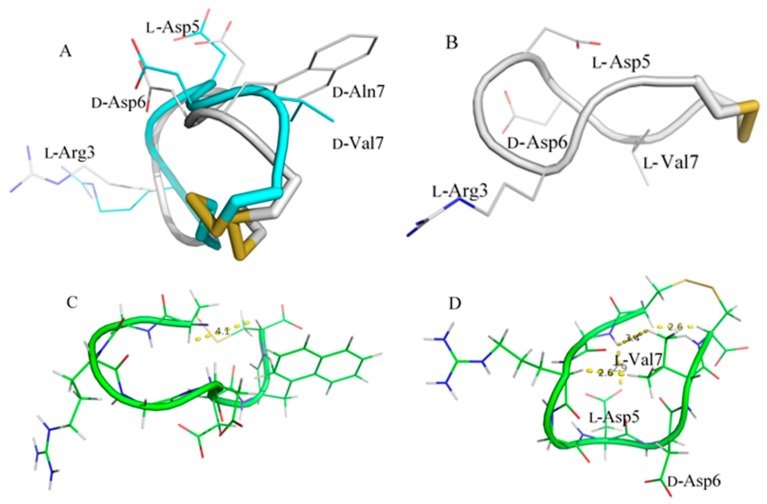
(**A**) Superimposed structures of LXW64 (grey) and LXW7 (cyan) with labeled side chains of L-Arg3, L-Asp5, D-Asp6 and D-Nal1 (X7) (LXW64) or D-Val7 (LXW7); (**B**) Sticks representation of the energy-minimized average structure of LXW11, L-Arg3, L-Asp5, D-Asp6, and D-Val7 are labeled; (**C**) The energy-minimized average structure of LXW64 with labeled distance between D-Cys1 H^α^ and D-Cys8 H^α^; (**D**) The energy-minimized average structure of LXW11 with labeled distances between D-Cys1 H^α^ and D-Cys8 H^α^, D-Val7 methyl protons and L-Gly2 H^N^/L-Arg3 H^α^, L-Asp5 carboxyl group, and L-Gly2 H^N^.

The structure of LXW64 is less compact and very similar to that of LXW7 since there is only one amino acid difference at position 7 [8]. The backbone RMSD of two peptides when aligned is 1.4 Å, and all side chains overlap very well (Figure 3A). Both peptides adopt an open ring-shaped structure, in which the side chains of two Asp residues point toward the outside ring plane, while Arg3 and *D*-Val7/*D*-Nal1 protrude from the ring structure in opposite directions. However, LXW64 and LXW7 are considerably different from LXW11, with a backbone RMSD of 2.5 Å after alignment. The bowl-like structures of LXW64 and LXW7 are twisted in LXW11, resulting in drastic changes of side-chain orientations of amino acids (Figure 3B). The side chains of Asp5 and *D*-Val7 in LXW11 are totally buried inside the circular structure, quite close to the opposite amino acids—Gly2 and Arg3. The side chain of *D*-Asp6 shows closer to the peptide backbone, and less protruding in comparison to LXW64 and LXW7 (Figure 3B). In addition, two cysteines (Cys1 and Cys8) in LXW11 are much closer to each other than that of LXW64 and LXW7 (Figure 3C,D), indicating that LXW11 is more structurally constrained than LXW64 and LXW7. These significant conformational differences are likely to cause dramatic changes in their binding affinities to αvβ3 integrin.

### 2.3. Complex Structure Models of LXW Peptides and αvβ3 Integrin

**Figure 4:**
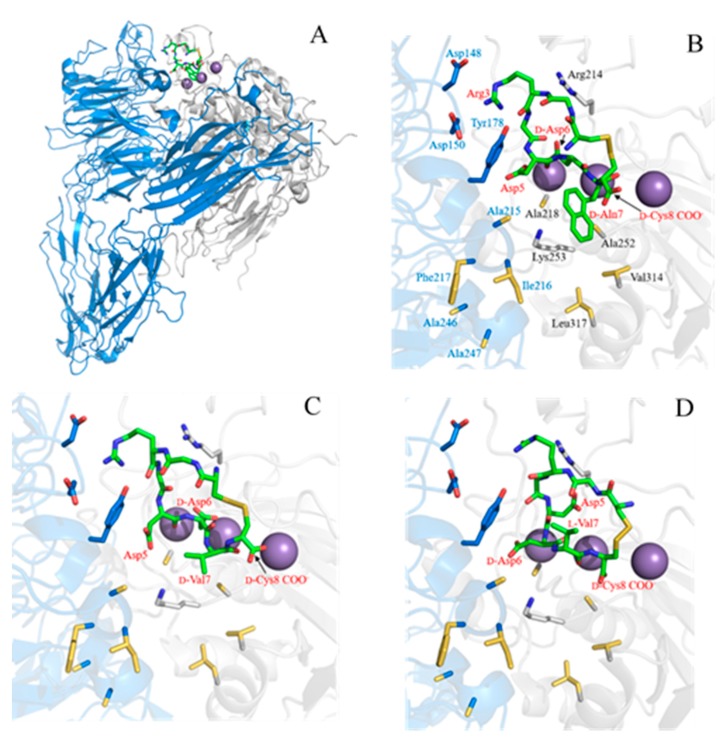
Docking models of LXW analogs (LXW64, LXW7 and LXW11) with αvβ3 integrin. (**A**) LXW64 (green) bound to αvβ3 integrin (α-subunit: blue, β-subunit: grey, Mn^2+^: purple); (**B**) Closeup view of LXW64 (green sticks) in the αvβ3 integrin binding site. Key amino acids are labeled with blue in α-subunit, black in β-subunit, and red in LXW64; (**C**) Closeup view of LXW7 (green sticks) in the αvβ3 integrin binding site. Key amino acids are highlighted with the same labels as in (**B,D**) Closeup view of LXW11 (green sticks) in the αvβ3 integrin binding site. Key amino acids are highlighted with the same labels as in Figure 4B.

To further examine the SARs of LXW analogs binding to the αvβ3 integrin, the computational models of three LXW analogs (LXW64, LXW7, LXW11) bound with the integrin were generated by means of docking calculation using AutoDock Vina [10] (see Materials and Methods for a detailed description), starting from the crystal structure of the extracellular segment of integrin αvβ3 in complex with cilengitide (PDB ID 1L5G). The energy-minimized average structures of LXW64, LXW7, and LXW11 were docked into the metal ion-dependent adhesion site (MIDAS), as shown in Figure 4A. In contrast to the crystal structure, the Arg3 side chain of LXW64 is able to form salt bridges with Asp150 and Asp148, but not with Asp218 in the complex model, indicating that Asp218 in the integrin is not optimal for LXW64. The carbonyl oxygen of Arg3 also forms a hydrogen bond with Tyr178 of the α-subunit. Both carboxylates of *D-*Asp6 side chain and *D-*Cys8 of LXW64 interact with Mn^2+^ ions within the MIDAS domain of the β-subunit. *D-*Asp6 is also close to Arg214 of the β-subunit and forms a salt bridge. In addition, a salt bridge is formed between LXW64 Asp5 and Lys253 of the β-subunit. Intriguingly, *D-Nal1 (X7)* side chain—naphthalene—is located in a potential hydrophobic pocket above Lys253 of the β-subunit, formed by nine hydrophobic amino acids from both α- (Ala215, Ile216, Phe217, Ala246, Ala247) and β-subunit (Val314, Leu317, Ala218, Ala252) adjacent to the MIDAS site (Figure 4B), which has never been reported. The binding pattern of LXW7 with the integrin is almost identical to LXW64 (Figure 4C). However, instead of only interacting with one of the carboxylate oxygens (Asp218 in α-subunit) in the previous docking simulation studies [7], LXW7 Arg3 is able to interact with both Asp148 and Asp150 in α-subunit (Figure 4C). *D-*Val7 side chain is also in the hydrophobic pocket, but much less extended than *D-Nal1* of LXW64, indicative of a weaker binding affinity. This is consistent with docking studies that LXW7 calculated free energy (−7.7 kcal/mol) is less than LXW64 (−9.0 kcal/mol). Although binding to the same site, LXW11 loses some critical interactions with the integrin compared to LXW64 and LXW7 (Figure 4D), even though the ionic interactions with Asp 148, Asp150, Lys253, Arg214 still remain. These missing interactions include the electrostatic interaction between Asp5 side-chain carboxylate and Mn^2+^ ions, the hydrophobic interaction between *D-*Val7 side chain and the hydrophobic groove as both are buried inside the circular structure. Furthermore, the configuration change from *D-*Cys (LXW64 and LXW7) to *L*-Cys in LXW11 alters the orientation of *L*-Cys8 carboxylate resulting in a much weaker interaction with Mn^2+^ ions. Its calculated free energy (−7.4 kcaL/moL) is much lower than other two peptides, indicating a significantly lower affinity binding (IC_50_). These results imply that the structures of LXW analog predominate their bindings with the αvβ3 integrin and a *D*-amino acid with a hydrophobic side chain at position 7 is preferred for a higher binding affinity.

### 2.4. New LXW-Analogous Peptide Screening by Autodock

**Figure 5:**
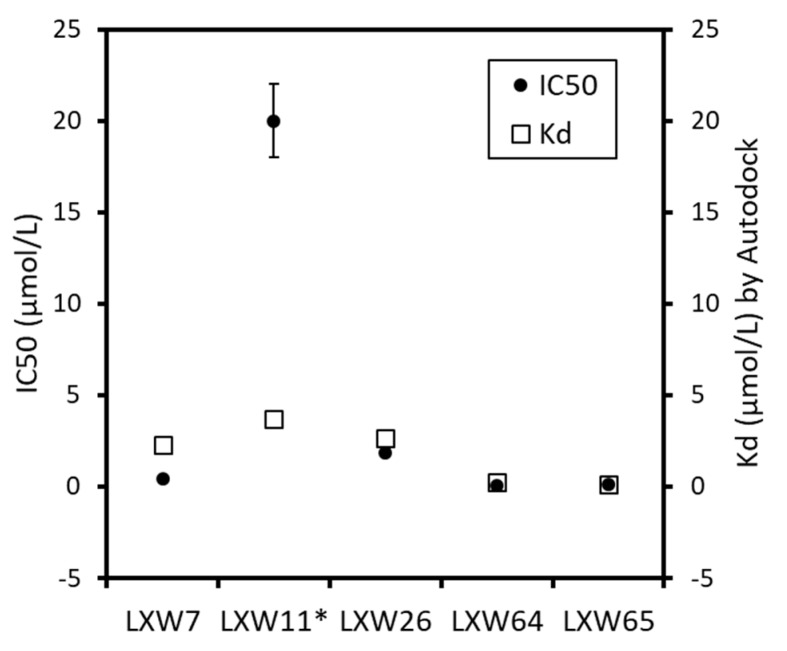
Comparison between the determined IC_50_ and Autodock-calculated Kd of representative LXW analogs.

To further develop more potent LXW-analogous peptides with a high affinity to αvβ3 integrin, we conducted in silico screening. Our strategy is to design new LXW64 mutants by replacing the X7 amino acid with hydrophobic residues, and then perform molecular docking by AutoDock Vina to screen new LXW analogs based on the docking scores. Five representative LXW analogs (LXW7, LXW11, LXW26, LXW64, LXW65) with known IC50s from previously in vitro screening were selected to test the feasibility of our strategy. Comparison between in vitro screening and in silico molecular docking (Table 3 and Figure 5) demonstrated a consistency within the results of both methods, suggesting that the in silico molecular docking can produce reliable results and is feasible for screening. We designed a series of LXW64 analogous peptides in which the X7 amino acid was replaced by D-amino acids in the SwissSidechain database (https://www.swisssidechain.ch/) [11]. In silico screening was performed by docking new designed LXW analogs to the crystal structure of αvβ3 integrin (PDB ID: 1L5G). Total 20 new LXW-analogous peptides were identified with a high binding affinity to the αvβ3 integrin referred to the new lead according to AutoDock prediction. All the new LXW analogs contain a X7 non-natural amino acid with either a cyclic (total 13) or non-cyclic (total 7) side chain as shown in Table 4. For these X7 amino acids, the side chains with cyclic structures were predicted with higher binding affinity than that of non-cyclic ones. Seven new LXW analogs with DNTL, DTRP, DPHE, DQ36, D5MW, D6MW, and DQX3 as the amino acid at position 7 were predicted with a binding affinity (Kd ≤ 0.5 μM) comparable to the new lead—LXW64 (0.25 μM). Intriguingly, one of the best X7 residues, 3-(9-anthryl)-*D*-alanine (DNTL) is structurally similar to the X7 of the new lead. This DNTL-containing LXW-analogous peptide (LXZ2) was predicted with a high binding affinity to the αvβ3 integrin.

### 2.5. In Vitro Examination of New LXW-Analogous Peptides

**Figure 6:**
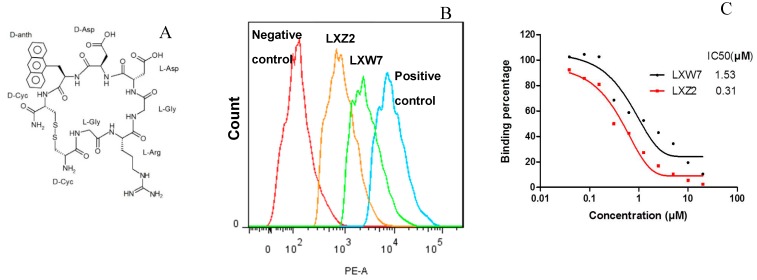
(**A**) Chemical structure of LXZ2; (**B**) Competitive binding assay. 5 μM of LXW7 (green) or LXZ2 (orange) competes with 1 μM biotinylated LXW7 binding to αvβ3-K562 cells, LXZ2 demonstrates a stronger binding affinity than LXW7. Positive control (cyan), cells were treated with 1μM biotinylated LXW7 and streptavidin-PE successively, and measured with flow cytometry. Negative control (red) represents cells without treatment of biotinylated LXW7. (**C**) IC50 measurement of LXW7 and LXZ2. The IC50s were determined with competitive binding assay by using a series of concentrations of LXW7 or LXZ2 competing with 1 μM biotinylated LXW7 binding to αvβ3-K562 cells.

To further verify our in silico screening results, we examined the binding of new LXW-analogous peptides to αvβ3 integrin in αvβ3-K562 cells. LXZ2 with DNTL as the X7 residue (Figure 6 left) was arbitrarily chosen and tested through flow cytometry for competing with 1 μM biotinylated LXW7 binding to αvβ3 integrin. As shown in Figure 6, LXZ2 exhibited a stronger binding affinity than the first lead—LXW7. The IC50 of the peptide inhibiting biotinylated LXW7 binding with K562/αvβ3+ cells was determined (Figure 6 right). Its converted IC50 (0.09 μmol/L referred to LXW7 in Table 3) is comparable to the new lead-LXW64 (0.07 μmol/L, Table 3) and the well-known RGD-cyclic pentapeptide ligand—cilengitide [5,8]. The result proved LXZ2 as a new αvβ3 integrin antagonist.

## 3. Discussion

Relatively rapid and low-cost identification of RGD-containing αvβ3 integrin antagonists The specificity of the integrins (e.g., αvβ3) recognizing and binding RGD motif has provided the molecular basis of integrin-targeted cancer therapy and enabled the development of several RGD-containing drugs for cardiovascular disease and cancer [2]. The first integrin antagonist—cilengitide— was discovered on the basis of “ligand-oriented design” via the optimization of RGD peptides by means of different chemical approaches including reduction of the conformational space by cyclization and spatial screening of cyclic peptides [12]. A similar RGD-based strategy combining with different techniques has led to identification of several RGD peptides, such as 1a-RGD [13], cyclopeptide c-Lys [14], LXW-analogous peptides [7,8]. These screening methods often require relatively expensive and time-consuming synthesis and a large amount of in vitro/in vivo cellular assays. Interestingly, two linear antagonists—RWr and RWrNM peptides—were identified recently using pharmacophore-based virtual screening [15], although cyclic peptides are likely preferred due to the fact that they usually show great biological activities compared to their linear counterparts because of their advantages including the conformational rigidity, the resistance to hydrolysis by exopeptidases, the receptor selectivity, and the efficient membrane-crossing property [16,17]. In our current studies, NMR structure determination of LXW64 and structural comparison with other LXW analogs, e.g., LXW7 and LXW11, enabled us to design new LXW-analogous peptides. Several new LXW-analogous antagonists were identified via in silico screening and one of the best LXW analogs—LXZ2—was arbitrarily chosen and verified by in vitro examination. Our results demonstrate that SAR studies provide clues for new RGD-containing αvβ3 integrin inhibitor design. Computational docking can predict the binding affinity quickly and narrow down the candidates; finally, the selected candidates can be tested by in vitro/in vivo examination. This provides a brief, rapid and relatively low-cost screening procedure for identification of αvβ3 integrin antagonists.

**Figure 7:**
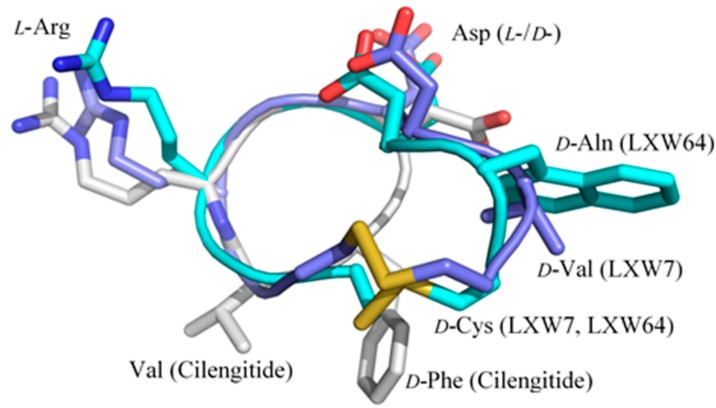
Structure superposition of LXW7 (light blue), LXW64 (cyan), and cilengitide from the crystal structure in complex with αvβ3 (PDB ID 1L5G, grey).

Structure–activity relationship between LXW peptides and αvβ3 LXW analogs are a new category of RGD-containing cyclopeptides that binds specifically to αvβ3 integrin [7,8]. The LXW analogs were designed and screened using OBOC combinatorial technology. Unlike other RGD cyclopeptide antagonists (i.e., cilengitide) [12,14], LXW analogs are extended circular structures with a disulfide bond formed between two cysteines in the peptide sequence (cGRGDdXc). *D-*configuration of two cysteines in these analogs is essential for the antagonistic activity, which can force the cyclopeptide to adopt an open bowl-shape conformation with the side chains of Arg, Asp, *D-*Asp and X7 residue pointing outwards from the peptide ring [8]. These protruding side chains are critical to interact with the integrin. LXW7 with *D-*Val7 was first identified as a lead ligand and further optimized by OBOC technology that led to the discovery of a 6-fold αvβ3-binding affinity increased antagonist—LXW64—with *D-Nal1* at position X7 [7,8]. As shown in Figure 7, LXW7 and LXW64 share similar structural patterns with an enlarged circular backbone structure, an extended hydrophobic moiety (disulfide-bonded cysteines and X7 residue) as well as an extra polar group (carboxyl group of *D-*Asp) in comparison with cilengitide. These unique structural properties suggest that LXW-analogous antagonists are more flexible and may adopt better fit-in conformations and exhibit better recognition specificity and selectivity than other RGD peptidomimetics (i.e., cyclic pentapeptide—cilengitide) when binding to αvβ3 integrin. In fact, LXW64 shows significant positive binding with αvβ3, weak or no binding to αvβ5, αIIbβ3, α5β1 expressed on K562 cells [8], while cilengitide can inhibit both αvβ3 and αvβ5 [18]. In addition, the biotinylated forms of LXW ligands show the similar binding strengths as LXW peptides against αvβ3 integrin [7], whereas biotinylated other RGD cyclopentapeptide ligands exhibit much weaker binding affinities than their free forms [7]. Thus, LWX analogous peptides are preferable as αvβ3 antagonists.

**Figure 8:**
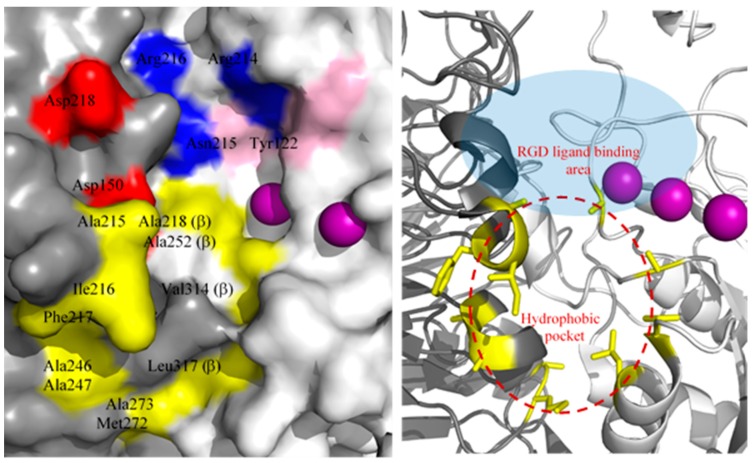
(**Left**): The RGD ligand-binding area on the major surface of αvβ3 integrin (PDB ID 1L5G) with α-subunit in grey, β-subunit in light grey, Mn^2+^ in purple. Asp150/Asp218 (red) in αv subunit form salt bridges with the arginine (R) guanidinium group of RGD ligand (i.e., cilengitide), whose aspartic acid (D) carboxy group contacts with Arg214 (blue), Asn215/Tyr122 (pink) in β3 subunit. The Arg216 is also involved in the contact with the ligand. A cluster of hydrophobic amino acids highlighted in yellow on the surface of α- and β-subunit form a hydrophobic circle near the RGD binding area, including Ala215, Ile216, Phe217, Ala246, Ala247, Met272 and Ala273 from α-subunit; Ala218, Ala252, Val314 and Leu317 from β-subunit; (**Right**): Closeup ribbon view of potential hydrophobic pocket (red dashed circle) at the major interface of αv (grey) and β3 (light grey) subunits, which is nearby RGD ligand-binding area (light blue). Hydrophobic amino acids (names labeled same as above) with side chains shown are highlighted in yellow.

**Figure 9:**
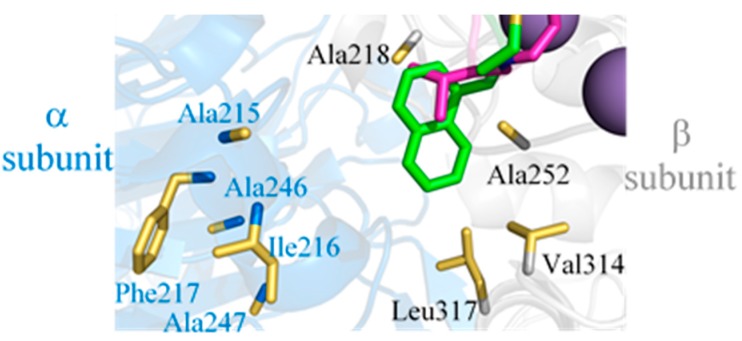
The hydrophobic amino acids (*D*-Val (purple), *D*-Nal1 (3-(1-naphthyl)-*D*-alanine, green)) at X7 position of LXW-analogous cyclic octapeptides (cGRGDd**X**c-NH_2_) binds at the major hydrophobic interface between the α (blue) and β (grey) subunits formed by Ala215, Ile216, Phe217, Ala246, Ala247 in the α subunit and Ala218, Ala252, Val314, Leu317 in the β subunit of the αvβ3 integrin.

Despite the structural similarity, LXW64 demonstrated 6-fold higher binding affinity than that of LXW7. This suggests that the hydrophobicity and aromaticity of X7 amino acid plays a critically important role in improving the binding affinity of LXW analogs. A careful inspection of crystal structure of αvβ3 in complex with RGD ligand (PDB ID 1L5G) shows that there is a potential hydrophobic pocket next to the RGD ligand-binding area; however, two hydrophobic amino acids of the RGD ligand—*D-*Phenylalanine and N-methyl-Valine—make no contact with this pocket, likely due to the compact and rigid structure of the small ring-shape ligand. The hydrophobic pocket is formed by hydrophobic amino acids from both α- and β-subunits as shown in Figure 8, which are located on the flexible surface area (e.g., flexible loops, short helical or β turn structures). As mentioned above, LXW-analogous peptides, especially LXW7 and LXW64 contain an extended hydrophobic moiety (i.e., disulfide-bonded *D-*cysteines and *D*-valine/3-(1-naphthyl)-*D*-alanine (*D*-Nal1)). It is highly possible that this hydrophobic moiety may undergo the hydrophobic interaction with this pocket and induce tertiary and quaternary structural changes of αvβ3 (Figure 9). In fact, LXW64 with a large polycyclic aromatic and hydrophobic X7 residue exhibited much higher binding affinity for the integrin than LXW7 (Figure 3A), suggesting the importance of hydrophobicity and aromaticity for the binding. It is surprising that this new hydrophobic pocket has not been identified previously [5], but likely plays a critical role in stabilizing the binding of LXW analogs to αvβ3 integrin and increase the binding affinity.

### Identification of a New LXW Analog—LXZ2

In our current study, 20 new LXW analogs were predicted via in silico screening with a high binding affinity to αvβ3 integrin. As shown in Table 4, all these peptides contain a hydrophobic non-natural amino acid at X7 position, which is consistent with our SAR studies. These X7 amino acids can be classified into two groups according to their side-chain structures—cyclic (total 13) and non-cyclic (aliphatic, total 7). All cyclic amino acids showed a lower but favorable Kd (≤ 1.0 μM) than aliphatic residues (Kd ≥ 1.0 μM). Among the cyclic amino acids, the aromatic (mainly polycyclic excepted DPHE) structures (e.g., DNTL, DTRP, DQ36, D5MW, DQX3, D6MW) showed a Kd ≤ 0.5 μM and were identified as the most favorable X7 residues, suggesting the importance of both hydrophobicity and aromaticity for the binding. However, further cellular binding assays are still needed to verify their bioactivities as potent integrin antagonists. It would not be surprising to discover that some peptides might not be functional as good as the docking prediction, likely resulting from the induced-fit effects [19]. We arbitrarily chose LXZ2 among all possible candidates (i.e., X7 = DPHE, DTRP, D5MW, D6MW, DQ36, DQX3) for further in vitro studies. LXZ2 was identified as a new αvβ3 antagonist. It has IC50 of 0.09 μM (this value was converted to match with the IC50s in Table 3), which is comparable to LXW64 (0.07 μM) [8] and the first antagonist—cilengitide—with IC50 of 0.25 μM [20]. In comparison with other RGD antagonists, LXZ2 as a LXW analog, not only shows a high binding affinity, but also likely exhibits the binding specificity against αvβ3 as previous studies showed that LXW peptides contain the auxiliary binding motifs including *D-*Asp at position 6 and the hydrophobicity of amino acid at X7 position [7,8]. LXZ2 contains non-proteinogenic amino acid—3-(9-Anthryl)-*D*-alanine—and its anthracene—tricyclic aromatic hydrocarbon—is noncarcinogenic, and readily biodegraded in soil and especially susceptible to degradation in the presence of light [21]. Considering the toxicity and environmental impact [22], LXZ2 can be used as a good substitute of LXW64 as both have a similar affinity to the integrin. Like LXW64, it may also be used as an excellent candidate vehicle for delivering drug-loaded nanoparticles for cancer imaging and therapy.

## 4. Materials and Methods

### 4.1. Synthesis of Peptides

All RGD-cyclic peptides, 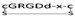 where a lower case letter represents *D*-amino acid (x is a variable amino acid) and a disulfide bridge is formed between two *D*-cysteines (*D*-Cys1 and *D*-Cys8), were chemically synthesized and purified by preparative RP-HPLC) from C S Bio Co (Menlo Park, CA, USA). For synthesis of LXW64 and LXZ2, the non-natural amino acids—Fmoc-3-(1-naphthyl)-*D*-alanine and Fmoc-3-(9-anthryl)-*D*-alanine—were purchased from Chem Impex (Wood Dale, IL, USA). HPLC purification was performed using an Agilent 1200 instrument and a Phenomenex Luna 5 μm C18(2) 100A 250 × 4.6 mm column. The peptides were eluted using a gradient of buffer A (0.1% TFA in water) and B (0.1% TFA in acetonitrile) with a flow rate of 1 mL/min. Each peptide eluted as a single peak via HPLC with > 97% purity, was verified by MS. The theoretical mass of LXW64 (918.98) was very close to the experimental value (918.73). For LXZ2, the experimentally measured mass of 968.72 was also similar to the expected 969.09. For NMR sample preparation, 20 mg peptides were dissolved in 0.6 mL DMSO-*d*_6_ solvent (Cambridge Isotope Laboratories, Inc., Tewksbury, MA, USA). The peptide solution was carefully transferred into an NMR tube after centrifugation. The repeated NMR analysis showed that the peptide samples were stable over several months in the chosen DMSO solvent.

### 4.2. NMR Spectroscopy

All NMR data were recorded at 295 K on a Bruker Ultrashield Plus 600 MHz spectrometer equipped with a 5 mm double resonance broad band room temperature probe (BBO) and a single-axis pulse-field-gradient accessory along the z-axis. All experiments were performed in a dimethyl sulfoxide solution to observe the amide protons. ^1^H-^1^H homonuclear two-dimensional (2D) NMR NOESY (τ_mix_ = 125 and 250 ms), TOCSY ((τ_mix_ = 70 ms), and DQF-COSY spectra were acquired using a sweep width of 14423 Hz and 1024 complex points in F1. The transmitter carrier was placed on the water resonance. Gradient heteronuclear correlation experiments, (^1^H-^13^C)-HMQC, (^1^H-^13^C)-HMBC, and (^1^H-^15^N)-HSQC were carried out to assign all carbon (^13^C) and nitrogen (^15^N) chemical shifts. The carbon carrier frequency was kept at 82 ppm for HMQC and 112 ppm for HMBC, the spectral width in the indirect carbon dimension was set to 150 ppm and 200 ppm for HMQC and HMBC, respectively. For ^1^H-^15^N HSQC, the nitrogen channel was centered at 116 ppm with a sweep width of 32 ppm. The States-TPPI method was used for quadrature detection in all indirectly detected dimensions. ^1^H, ^13^C, and ^15^N NMR chemical shifts were reported using DSS and DMSO-*d*_6_ as references. NMR data were processed by NMRPipe [23] and analyzed with SPARKY (https://www.cgl.ucsf.edu/home/sparky/) [24].

### 4.3. Experimental Constraints and Structure Calculation

The NMR structures of LXW64 were calculated based on NOE distances and dihedral angle restraints. Distance constraints were extracted from 2D NOESY recorded using two mixing times (125 ms and 250 ms). NOE cross peaks (NOEs) were classified into strong, medium and weak according to the intensities, and assigned to the interproton distances of 2.9, 3.5 and 5 Å. The upper bound distance constraints of the NOEs involving methyl and methylene groups were modified using pseudoatom correction [25]. The backbone dihedral angles (φ) were calculated from the Karplus Equation using ^3^*J*_HNα_ coupling constants measured from DQF-COSY spectrum [26]. For side-chain dihedral angles, the χ1 was defined according to the NOE intensities between the amino proton (H^N^) and two β protons (H^β^) in comparison with the NOE intenwrsities between α proton (H^α^) and two β protons in the same residue [27,28]. The additional constraint data from the chemical shifts of C^α^, C^β^ and H^α^ were used in the final structure refinement. The NMR-derived distances and dihedral angles then served as constraints for calculating the three-dimensional structures using distance geometry and restrained molecular dynamics. Structure calculations were performed using the YASAP protocol within X-PLOR version 2.36 [29,30] installed on a regular Linux workstation (CentOS 6), as described previously [31]. Fifty independent structures were calculated, and the 10 lowest-energy structures were selected. The average total and experimental distance energy were 185 ± 7 and 12 kcal/mol. Ramachandran analysis of the determined structures was performed through MolProbity [32] with default settings to measure the structure reliability. The average root-mean-square (rms) deviation from an idealized geometry for bonds and angles were 0.0094 Å and 2.27°. None of the distance and angle constraints were violated by more than 0.4 Å and 4°, respectively.

### 4.4. Complex Modeling and Autodock Screening

Complex modeling of three representative LXW analogs (LXW7, LXW11, LXW64) and in silico screening of new LXW analogs were performed using the program AutoDock 4.20 [10] by following stepwise guidelines [33]. The NMR structures of LXW7 and LXW11 determined previously [8], and of LXW64 were used for docking. For screening of new RGD-containing peptides, all peptide mutants were generated using PyMol Mutagenesis Wizard by substituting *D*-Val7 of LXW7 with non-native amino acids in the SwissSidechain database (https://www.swisssidechain.ch/) [11]. Each peptide was prepared by the AutoDock Tools GUI (graphical user interface) and with the rotatable side-chain bonds set to allow rotation and docked into the ligand-binding pocket of integrin αvβ3 (PDB ID 1L5G) [5], in which the default ligand was removed using a text editing software. The modified αvβ3 structure was prepared for AutoDock Vina [34] compatibility with AutoDock Tools GUI [10]. The grid box with spacing size of 0.375 Å was placed in the center of the binding pocket in accordance with the ligand (cyclo(RGDf-N(Me))V-) on the crystal structure (PDB ID 1L5G). The grid box center was selected to include all the residues in the binding pocket and set as follows: X-center 18.792, Y-center 42.057, and Z-center 43.65. The configuration settings for AutoDock Vina were set to default except for the number of binding modes, which was set to 8. The appropriate search space parameters were determined through eBoxSize for each ligand [35]. A stochastic Lamarckian genetic algorithm was employed for computing peptide conformations within the active pocket by using the default parameters. A total of 1000 conformers were generated for the ligand in the binding pocket, which were clustered using a 2.0 Å root-mean-square deviation. The output ligand files were modified to contain only the optimal configuration through UCSF Chimera version 1.9 [36]. The receptor-ligand complexes were developed through the UCSF Chimera ViewDock extension and prepared using the FindHBond tool. The receptor and ligand-binding modes were joined using a text editing software and analyzed by PyMol 1.7 (open-source) [37]. The AutoDock screening generates an energy score (kcal/moL), which represents the free energy (ΔG) of peptide binding to integrin αvβ3. The peptide binding affinity (Kd) is obtained according to calculation of Ki in AutoDock 4 at 298K.

### 4.5. Flow Cytometry

The human leukemia cancer cells (K562, ATCC, Manassas, VA, USA) transfected with αvβ3-integrin were grown in the RPMI1640 medium and used for the assay as previously described [7,8]. To demonstrate the peptides binding to the integrin and compare with the binding affinity of LXW7, the cells in each sample were incubated with 1.0 μM biotinylated LXW7 in 50 μL of phosphate-buffered saline (PBS) containing 10% fetal calf serum (FBS) and 1 mM MnCl_2_ for 30 min on ice. The samples were washed with 1 mL PBS containing 1% FBS for three times, then incubated with a 1:500 dilution of streptavidin-PE (1 mg/mL) for 30 min on ice followed by a single wash with 1 mL of PBS containing 1% FBS. Finally, the samples were analyzed via flow cytometry (Coulter XL-MCL). To measure the half-maximal inhibitory concentration (IC50) of the peptides, various diluted peptide solutions were mixed with 1.0 μM biotinylated LXW7, then incubated with cells, and followed by streptavidin-PE incubation. The samples were run through flow cytometer and Mean Fluorescence Intensity was decided for each individual sample. The IC50s were calculated from each sample based on the readings.

## 5. Conclusions

In this study, SARs of LXW-analogous cyclic octapeptides and αvβ3 integrin had been investigated through NMR structure determination and complex modeling. The hydrophobicity and aromaticity of the X7 amino acid in LXW-analogous sequence was found to be important for enhancing LXW analogs binding to the integrin, likely through the interaction with a potential hydrophobic pocket on the integrin surface. The SAR studies led to the identification of several new LXW-analogous peptides by in silico screening, which were predicted with high binding affinity. One of the best peptides—LXZ2—was arbitrarily chosen and verified by cell-based competitive binding assays and found to be comparable to other well-known “head-to-tail” RGD cyclopeptide—LXW64—and cilengitide. Most importantly, this new αvβ3 antagonist was identified through a brief and comparatively inexpensive screening procedure, benefited from the SAR studies. LXZ2 as an analogous peptide of LXW64 demonstrated a high binding affinity to αvβ3 integrin transfected in K562 cells, can be used a vehicle for delivery of cytotoxic payload to tumors and tumor blood vessels with overexpressing αvβ3 integrin.

## Figures and Tables

**Table 1:** Representative LXW analogs and their IC_50_s *.

Peptide	Amino Acid Sequence ^#^	IC_50_ (μmoL/L)
LXW7	cGRGDdvc-NH_2_	0.46
LXW11	CGRGDdvC-NH_2_	>20
LXW64	cGRGDd-*D*Nal1-c-NH_2_	0.07

* IC_50_ of the peptide is the concentration of peptide required for inhibition of 0.5 µmoL/L biotinylated LXW7 binding to K562/αvβ3+ cells by half. ^#^ The lowercase letters indicate *D*-amino acids, whereas the uppercase letters denote *L*-amino acids.

**Table 2:** Structure Statistics for the Ensembles of 10 Calculated Structures of LXW64.

NOE Restraints (Total)	58
dihedral angle restraints	14
RMSD from ideal geometry	
bond length (Å)	0.0094 ± 0.00041
bond angles (degree)	2.27 ± 0.04
Ramachandran plot	
allowed region (%)	100
disallowed region (%)	0
RMSD of atom position from average structure	
main chain (Å)	0.34 ± 0.072
non-hydrogen (Å)	1.47 ± 0.16

**Table 3:** The experimentally measured half-maximal inhibitory concentration (IC_50_) and computationally calculated binding free energy (kcal/mol) and equilibrium dissociation constant (Kd) by Autodock of LXW analogs binding to αvβ3 integrin.

RGD Peptide	X Amino Acids in LXW Analogs (CGRGDdXc-NH_2_)	IC_50_ (µmoL/L)	Binding Free Energy (kcaL/moL)	Kd (µmoL/L) by Autodock ^#^
LXW7	D-Val	0.46	−7.7	2.27
LXW11 *	D-Val and L-Cys	>20	−7.4	3.70
LXW26	D-Ile	1.84	−7.6	2.67
LXW64	D-Nal1	0.07	−9.0	0.24
LXW65	D-Nal2	0.13	−9.3	0.15

* two cysteines are L-configuration. **^#^** According to calculation of Ki in Autodock 4.

**Table 4:** 20 X7 amino acids screened from the SwissSidechain database (https://www.swisssidechain.ch/) in LXW-analogous cyclic octapeptides (cGRGDd**X**c-NH_2_) and the peptides’ binding affinities (Kd, µmoL/L) to αvβ3 integrin calculated by Autodock 4.2. (note: Residues highlighted in bold are comparable to LXW64 listed as a reference and all non-natural amino acid codes are from the SwissSidechain database).

Peptide/X_7_ Residue	Kd (µmoL/L)	Peptide/X_7_ Residue	Kd (µmoL/L)	Peptide/X_7_ Residue	Kd (µmoL/L)
DNAL1 (LXW64) 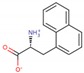	0.25	DCPE 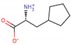	0.70	DNLE 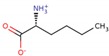	1.63
**DNTL (LXZ2)** 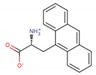	**0.29**	DHL1 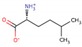	1.38	DNVA 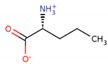	1.16
**DTRP** 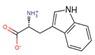	**0.30**	DALC 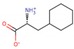	0.50	DQ33 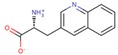	0.50
DPZ4 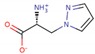	0.59	DLVG 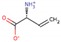	0.83	**DQ36** 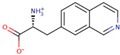	**0.30**
DLEU 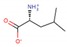	1.16	**D5MW** 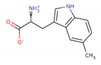	**0.30**	**DQX3** 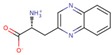	**0.38**
**DPHE** 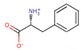	**0.36**	**D6MW** 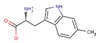	**0.42**	DTH9 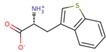	0.59
DMET 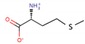	0.83	D2TH 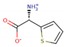	0.98	DAHP 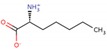	1.63

## Supplementary Materials

Supplementary materials can be found at https://www.mdpi.com/1422-0067/21/9/3076/s1.

## Abbreviations

SAR	Structure–Activity Relationship
RGD	Arginylglycylaspartic acid
NMR	Nuclear Magnetic Resonance
NOE	Nuclear Overhauser Effect
HSQC	Heteronuclear Single Quantum Correlation
TOCSY	TOtal Correlated SpectroscopY
NOESY	Nuclear Overhauser Effect Spectroscopy
HMQC	Heteronuclear Multiple-Quantum Correlation
HMBC	Heteronuclear Multiple Bond Correlation
RMSD	Root-Mean-Squared Derivation
RP-HPLC	Reversed Phase-High Performance Liquid Chromatography
MS	Mass spectrometry
DMSO	Dimethyl sulfoxide
DSS	2,2-Dimethyl-2-silapentane-5-sulfonate
PBS	Phosphate-Buffered Saline
FBS	Fetal Calf Serum
IC50	Half-Maximal Inhibitory Concentration
