# Supplementary material for: Structure–Activity Relationship of RGD-Containing Cyclic Octapeptide and αvβ3 Integrin Allows for Rapid Identification of a New Peptide Antagonist
Source: Int J Mol Sci. 2020 Apr 27;21(9):3076. doi: 10.3390/ijms21093076 (PMC7246635; doi:10.3390/ijms21093076)
Supplement: Supplementary file 1 [file ijms-21-03076-s001.pdf]

Table S1. ^{1}H, ^{13}C and ^{15}N Chemical Shifts of LXW64 (**cGRGDd-D-Aln-c**) at 22 °C, DMSO-d_6

Res#	^{1}H				^{15}N	^{13}C			
	NH	αH	βH	Others		C′	αC	βC	Others
c1	8.42	4.29	3.15/3.26		119.22	169.85	54.37	41.73	
G2	8.88	4.08/3.77			109.51	170.89	44.98		
R3	8.35	4.30	1.53/1.72	1.48/1.42(γH)<br>3.09(δH)<br>7.73(εNH)<br>7.35/7.17(ηNH)	119.57	174.21	55.12	31.58	27.69(γC)<br>43.02(δC)<br>159.22(ζC)
G4	8.20	3.92/3.68			106.42	171.77	44.66		
D5	8.34	4.54	2.48/2.59		116.94	173.81	52.58	38.86	173.81(γC)
d6	8.28	4.40	2.44/2.54		118.06	170.04	52.96	38.69	174.04(γC)
aln7<br>(*D*-<br>Aln)	7.98	4.65	3.32/3.56	7.39(δH)<br>7.42(εH)<br>7.81(ζH)<br>8.19/7.93(ηH)<br>7.60/7.54(θH)	116.19	174.03	56.38	36.94	135.29(γC)<br>130.04(δC)<br>128.13(εC)<br>129.80(ζC)<br>126.30/131.33(ηC)<br>128.82/128.33(θC)
c8	8.40	4.51	2.97/3.15		118.08	174.16	54.65	42.87	

S2. LXW64 Coordinates

REMARK final LXW64 structures

ATOM 1 CA CYS 1 9.736 -2.438 -2.605 1.00 0.65

ATOM 2 HA CYS 1 8.974 -3.029 -3.108 1.00 0.94

ATOM 3 HB1 CYS 1 10.329 -0.672 -3.650 1.00 0.65

ATOM 4 HB2 CYS 1 9.814 -0.392 -2.008 1.00 1.07

ATOM 5 C CYS 1 9.570 -2.622 -1.103 1.00 0.53

ATOM 6 O CYS 1 10.296 -2.046 -0.317 1.00 0.64

ATOM 7 CB CYS 1 9.589 -0.942 -2.910 1.00 0.44

ATOM 8 SG CYS 1 7.975 -0.375 -3.503 1.00 0.78

ATOM 9 N CYS 1 11.072 -2.907 -3.047 1.00 1.10

ATOM	10	N	GLY	2	8.610	-3.425	-0.735	1.00	0.41
ATOM	11	HN	GLY	2	8.050	-3.864	-1.407	1.00	0.47
ATOM	12	CA	GLY	2	8.370	-3.667	0.715	1.00	0.32
ATOM	13	HA1	GLY	2	7.321	-3.874	0.874	1.00	0.38
ATOM	14	HA2	GLY	2	8.643	-2.781	1.272	1.00	0.43
ATOM	15	C	GLY	2	9.204	-4.849	1.214	1.00	0.16
ATOM	16	O	GLY	2	10.401	-4.732	1.378	1.00	0.27
ATOM	17	N	ARG	3	8.563	-5.966	1.442	1.00	0.17
ATOM	18	HN	ARG	3	7.595	-6.026	1.296	1.00	0.28
ATOM	19	CA	ARG	3	9.330	-7.143	1.929	1.00	0.29
ATOM	20	HA	ARG	3	9.919	-6.822	2.785	1.00	0.37
ATOM	21	CB	ARG	3	8.342	-8.261	2.319	1.00	0.55
ATOM	22	HB1	ARG	3	7.900	-8.032	3.278	1.00	0.49
ATOM	23	HB2	ARG	3	8.874	-9.199	2.395	1.00	0.97
ATOM	24	CG	ARG	3	7.235	-8.386	1.263	1.00	0.86
ATOM	25	HG1	ARG	3	7.620	-8.091	0.297	1.00	1.23
ATOM	26	HG2	ARG	3	6.412	-7.738	1.527	1.00	1.16
ATOM	27	CD	ARG	3	6.745	-9.844	1.200	1.00	1.46
ATOM	28	HD1	ARG	3	7.421	-10.484	1.751	1.00	1.66
ATOM	29	HD2	ARG	3	6.701	-10.171	0.170	1.00	2.13
ATOM	30	NE	ARG	3	5.381	-9.932	1.807	1.00	2.12
ATOM	31	HE	ARG	3	5.148	-9.365	2.568	1.00	2.46

ATOM	32	CZ	ARG	3	4.500	-10.774	1.326	1.00	2.85
ATOM	33	NH1	ARG	3	3.228	-10.484	1.429	1.00	3.51
ATOM	34	HH11	ARG	3	2.947	-9.632	1.870	1.00	3.55
ATOM	35	HH12	ARG	3	2.536	-11.111	1.071	1.00	4.25
ATOM	36	NH2	ARG	3	4.922	-11.876	0.768	1.00	3.42
ATOM	37	HH21	ARG	3	5.901	-12.065	0.715	1.00	3.44
ATOM	38	HH22	ARG	3	4.265	-12.534	0.395	1.00	4.14
ATOM	39	C	ARG	3	10.295	-7.682	0.873	1.00	0.27
ATOM	40	O	ARG	3	11.464	-7.349	0.877	1.00	0.61
ATOM	41	N	GLY	4	9.797	-8.498	-0.014	1.00	0.27
ATOM	42	HN	GLY	4	8.847	-8.741	0.013	1.00	0.56
ATOM	43	CA	GLY	4	10.691	-9.059	-1.071	1.00	0.22
ATOM	44	HA1	GLY	4	10.266	-9.985	-1.433	1.00	0.30
ATOM	45	HA2	GLY	4	11.662	-9.262	-0.644	1.00	0.21
ATOM	46	C	GLY	4	10.851	-8.092	-2.246	1.00	0.19
ATOM	47	O	GLY	4	9.882	-7.561	-2.754	1.00	0.41
ATOM	48	N	ASP	5	12.075	-7.882	-2.660	1.00	0.29
ATOM	49	HN	ASP	5	12.831	-8.331	-2.225	1.00	0.49
ATOM	50	CA	ASP	5	12.306	-6.955	-3.799	1.00	0.32
ATOM	51	HA	ASP	5	12.535	-5.966	-3.411	1.00	0.40
ATOM	52	CB	ASP	5	13.456	-7.528	-4.642	1.00	0.56
ATOM	53	HB1	ASP	5	14.402	-7.332	-4.156	1.00	0.70

ATOM	54	HB2	ASP	5	13.457	-7.073	-5.621	1.00	0.60
ATOM	55	CG	ASP	5	13.267	-9.040	-4.787	1.00	0.69
ATOM	56	OD1	ASP	5	14.285	-9.713	-4.817	1.00	1.18
ATOM	57	OD2	ASP	5	12.115	-9.434	-4.859	1.00	1.41
ATOM	58	C	ASP	5	11.059	-6.876	-4.670	1.00	0.19
ATOM	59	O	ASP	5	10.786	-7.774	-5.445	1.00	0.37
ATOM	60	N	ASP	6	10.322	-5.805	-4.533	1.00	0.17
ATOM	61	HN	ASP	6	10.576	-5.103	-3.899	1.00	0.29
ATOM	62	CA	ASP	6	9.091	-5.675	-5.356	1.00	0.31
ATOM	63	HA	ASP	6	8.242	-5.561	-4.689	1.00	0.48
ATOM	64	CB	ASP	6	8.976	-6.958	-6.184	1.00	0.51
ATOM	65	HB1	ASP	6	9.871	-7.095	-6.773	1.00	0.53
ATOM	66	HB2	ASP	6	8.120	-6.901	-6.838	1.00	0.68
ATOM	67	CG	ASP	6	8.813	-8.145	-5.230	1.00	0.58
ATOM	68	OD1	ASP	6	8.541	-7.873	-4.071	1.00	1.30
ATOM	69	OD2	ASP	6	8.967	-9.255	-5.713	1.00	1.01
ATOM	70	C	ASP	6	9.132	-4.477	-6.307	1.00	0.20
ATOM	71	O	ASP	6	9.570	-3.403	-5.945	1.00	0.23
ATOM	72	N	ALN	7	8.664	-4.696	-7.507	1.00	0.19
ATOM	73	HN	ALN	7	8.318	-5.582	-7.742	1.00	0.29
ATOM	74	CA	ALN	7	8.651	-3.605	-8.520	1.00	0.19
ATOM	75	HA	ALN	7	7.683	-3.626	-9.015	1.00	0.30

ATOM	76	CB	ALN	7	9.795	-3.844	-9.524	1.00	0.35
ATOM	77	HB1	ALN	7	9.659	-3.199	-10.380	1.00	1.21
ATOM	78	HB2	ALN	7	9.771	-4.873	-9.853	1.00	1.21
ATOM	79	CG	ALN	7	11.167	-3.554	-8.897	1.00	1.15
ATOM	80	CD1	ALN	7	12.187	-3.071	-9.706	1.00	2.00
ATOM	81	CD2	ALN	7	11.397	-3.782	-7.555	1.00	1.51
ATOM	82	HD2	ALN	7	10.602	-4.157	-6.928	1.00	0.89
ATOM	83	CE1	ALN	7	13.448	-2.818	-9.154	1.00	3.23
ATOM	84	CE2	ALN	7	12.633	-3.535	-7.014	1.00	2.68
ATOM	85	HE2	ALN	7	12.809	-3.716	-5.964	1.00	2.96
ATOM	86	CZ	ALN	7	13.648	-3.059	-7.805	1.00	3.54
ATOM	87	HZ	ALN	7	14.617	-2.871	-7.367	1.00	4.48
ATOM	88	CH1	ALN	7	11.985	-2.829	-11.056	1.00	1.73
ATOM	89	HH1	ALN	7	11.015	-3.021	-11.490	1.00	0.87
ATOM	90	CH2	ALN	7	14.453	-2.336	-9.977	1.00	4.09
ATOM	91	HH2	ALN	7	15.430	-2.140	-9.561	1.00	5.03
ATOM	92	CJ1	ALN	7	12.995	-2.350	-11.856	1.00	2.61
ATOM	93	HJ1	ALN	7	12.814	-2.168	-12.907	1.00	2.40
ATOM	94	CJ2	ALN	7	14.229	-2.103	-11.318	1.00	3.78
ATOM	95	HJ2	ALN	7	15.026	-1.726	-11.944	1.00	4.47
ATOM	96	C	ALN	7	8.813	-2.232	-7.886	1.00	0.17
ATOM	97	O	ALN	7	7.872	-1.465	-7.819	1.00	0.24

ATOM	98	N	CYS	8	9.996	-1.939	-7.434	1.00	0.25
ATOM	99	HN	CYS	8	10.730	-2.584	-7.505	1.00	0.26
ATOM	100	CA	CYS	8	10.214	-0.615	-6.807	1.00	0.41
ATOM	101	HA	CYS	8	10.890	-0.732	-5.965	1.00	0.48
ATOM	102	HB1	CYS	8	8.280	0.159	-7.256	1.00	1.16
ATOM	103	HB2	CYS	8	8.998	0.833	-5.817	1.00	1.09
ATOM	104	CB	CYS	8	8.845	-0.084	-6.369	1.00	0.49
ATOM	105	SG	CYS	8	7.821	-1.171	-5.352	1.00	1.40
ATOM	106	C	CYS	8	10.810	0.375	-7.802	1.00	0.52
ATOM	107	OT1	CYS	8	10.537	1.550	-7.622	1.00	1.28
ATOM	108	OT2	CYS	8	11.502	-0.098	-8.688	1.00	1.12

END
